# Mobile genetic elements in the pathogenesis of human reproductive disorders

**DOI:** 10.18699/vjgb-26-69

**Published:** 2026-07

**Authors:** A.A. Babovskaya, E.A. Trifonova, V.A. Stepanov

**Affiliations:** Research Institute of Medical Genetics, Tomsk National Research Medical Center of the Russian Academy of Sciences, Tomsk, Russia; Research Institute of Medical Genetics, Tomsk National Research Medical Center of the Russian Academy of Sciences, Tomsk, Russia; Research Institute of Medical Genetics, Tomsk National Research Medical Center of the Russian Academy of Sciences, Tomsk, Russia

**Keywords:** mobile genetic elements, transposons, reproduction, placenta, human diseases, мобильные элементы генома, транспозоны, репродукция, плацента, заболевания человека

## Abstract

Mobile genetic elements (MGEs), or transposons, are autonomous DNA sequences capable of moving and proliferating within the genome. Long considered “selfish” or “junk” DNA, MGEs are now recognized as key components involved in genome evolution, the regulation of gene expression, and the pathogenesis of various diseases. In humans, MGEs are divided into two main classes: retrotransposons (Class I), which replicate via an RNA intermediate through a “copy-and-paste” mechanism, and DNA transposons (Class II), which move via a “cut-and-paste” mechanism without an RNA intermediate. According to the Human Genome Project, retrotransposons constitute the majority (approximately 42 %) of the MGE fraction within the human genome. The most abundant are the non-long terminal repeat (non-LTR) retrotransposons, dominated by autonomous LINE-1 elements. Although approximately 500,000 LINE-1 copies are present in the genome, the vast majority are defective, and only a small fraction (<100) retain the capacity for transposition in modern humans. The second most prevalent group (about 10.6 %) within the retrotransposon family is the short interspersed nuclear elements (SINEs), specifically Alu elements, which are non-autonomous and hijack the LINE-1 molecular machinery for their mobilization and integration. MGE activity is a tightly regulated process in somatic tissues. Epigenetic mechanisms, particularly DNA methylation, normally effectively suppress MGE expression and mobility. Disruption of this control is associated with a wide range of pathologies. For instance, hypomethylation and reactivation of retrotransposons, notably LINE-1, have been demonstrated in various cancers, as well as in neurodegenerative and autoimmune diseases. The aim of this review is to provide a systematic analysis of the current understanding of the role of mobile genetic elements, particularly LINE-1, Alu, and HERV retrotransposons, in the development of human reproductive system disorders. This also includes diseases associated with placental pathology, an area that remains insufficiently studied to date, despite a growing body of data.

## Abstract (ru)

Мобильные генетические элементы (МГЭ), или транспозоны, представляют собой автономные последовательности ДНК, способные к перемещению и распространению в пределах генома. Долгое время рассматривавшиеся как «эгоистичная» или «мусорная» ДНК в настоящее время МГЭ считаются одними из компонентов, принимающих участие в эволюции генома, регуляции экспрессии генов и патогенезе ряда заболеваний. У человека МГЭ подразделяются на два основных класса: ретротранспозоны (I класс), реплицирующиеся через промежуточную стадию РНК по механизму «копирования–вставки» (copy-and-paste), и ДНК-транспозоны (II класс), перемещающиеся по механизму «вырезания–вставки» (cut-and-paste) без РНК-интермедиата. Ретротранспозоны, по данным проекта «Геном человека», составляют примерно 42 % от доли МГЭ в человеческом геноме. Наиболее многочисленны ретротранспозоны без длинных концевых повторов (non-LTR), среди которых доминируют автономные элементы LINE-1. Несмотря на то что в геноме присутствует около 500 000 копий LINE-1, большинство из них дефектно и лишь малая часть (<100) сохраняет возможность к транспозиции у современного человека. Второй по распространенности группой (около 10.6 %) семейства ретротранспозонов являются короткие диспергированные повторы Alu (SINE-элементы), которые, будучи неавтономными, используют для перемещения и встраивания молекулярный аппарат LINE- 1. Активность МГЭ – строго регулируемый процесс в соматических тканях. Эпигенетические механизмы, в частности метилирование ДНК, в норме эффективно подавляют экспрессию и мобильность мобильных генетических элементов. Нарушение этого контроля ассоциировано с широким спектром патологий. Так, гипометилирование и реактивация ретротранспозонов, в особенности LINE-1, продемонстрированы при различных типах рака, нейродегенеративных и аутоиммунных заболеваниях. Цель настоящего обзора – систематический анализ современных представлений о роли мобильных генетических элементов, в частности ретротранспозонов LINE-1, Alu и HERV, в развитии заболеваний репродуктивной системы человека, также включая заболевания, связанные с патологией плаценты, область которых остается недостаточно изученной, несмотря на растущий объем данных.

## Introduction

**Fig. 1.**
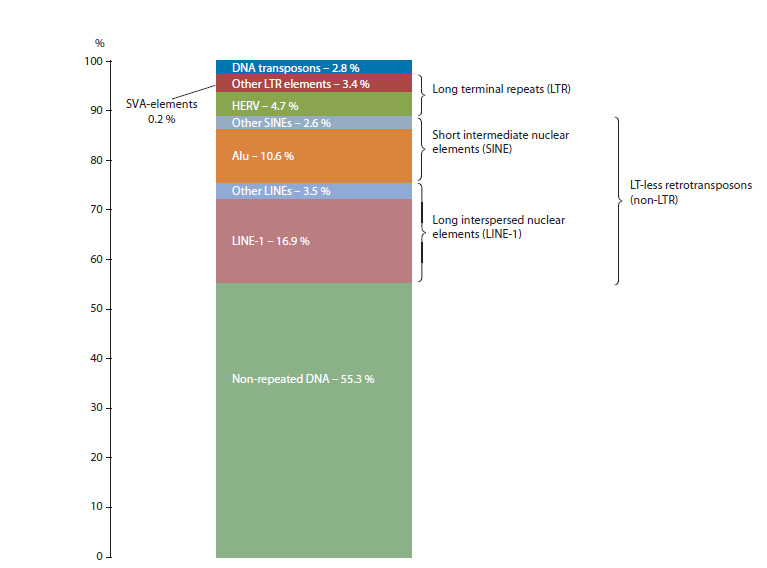
Proportions of mobile genetic elements (MGEs) in the human genome and their distribution among different families and subfamilies according to (Lander et al., 2001).

Contemporary research on mobile genetic elements (MGEs) and their role in disease development represents a rapidly advancing field of molecular biology and genetics. In humans, MGEs are classified into two major classes: retrotransposons, which replicate via an mRNA intermediate through a “copyand- paste” mechanism, and DNA transposons, which utilize a DNA segment as an intermediate during transposition. The most abundant group in the human genome consists of retrotransposons, which are further subdivided into elements containing long terminal repeats (LTRs) and those lacking LTRs (non-LTR elements). The latter predominate, accounting for approximately 75.2 % of all mobile elements and about 33.6 % of the entire genome (Lander et al., 2001; Chénais et al., 2012). Among non-LTR retrotransposons, long interspersed nuclear elements-1 (LINE-1; Fig. 1) have undergone extensive expansion, reaching approximately 500,000 copies in the human genome, the majority of which are represented by defective or fragmented sequences. The LINE-1 family accounts for about 38 % of all MGEs and 16.9 % of the genome (Fig. 1). However, only a small fraction of LINE-1 copies retain transpositional activity, that is, the capacity for autonomous retrotransposition via reverse transcription of their mRNA and integration of the resulting cDNA into new genomic loci.

The second most abundant category of MGEs comprises non-autonomous short interspersed nuclear elements (SINEs), particularly Alu repeats, which constitute approximately 24 % of all MGEs and 10.6 % of the genome. The human reference genome contains around one million copies of Alu elements derived from several evolutionarily young subfamilies (e. g., AluY). Their extensive amplification is associated with a burst of retrotranspositional activity in ancestral primates approximately 35–40 million years ago. For their retrotransposition, Alu elements rely on the enzymatic machinery of LINE-1 elements, including endonuclease and reverse transcriptase. Other representatives of SINEs in humans include MIR repeats (Mammalian-wide Interspersed Repeats). In addition, composite SVA elements composed of homologous SINE regions, variable number tandem repeats (VNTRs), and Alu-like sequences also belong to the group of nonautonomous elements mobilized by LINE-1. Only certain subfamilies are considered transpositionally active in the modern human genome, including Alu Ya5, Alu Yb8, Alu Yb9, SVA-E, and SVA-F. LINE-1 activity in the germ line leads to the emergence of new LINE-1, Alu, and SVA insertions, which serve as population genetic markers (Khitrinskaya et al., 2003, 2014).

LTR retrotransposons are present in smaller numbers in the human genome and are mainly represented by human endogenous retroviruses (HERVs). In terms of their structure and mechanism of retrotransposition, HERVs are similar to retroviruses but have lost functional envelope (env) genes. The activity of LTR retrotransposons in humans has markedly declined over the past several million years; nevertheless, their sequences still constitute approximately 8 % of the genome (Fig. 1). The classification of HERVs is based on the type of tRNA used to initiate reverse transcription; the evolutionarily youngest and most active subfamily is HERV-K (which uses lysine tRNA). Other groups include HERV-I (isoleucine tRNA) and HERV-L (leucine tRNA). Class II elements (DNA transposons) are considerably less common in the human genome, accounting for only about 6 % of all MGEs and 2.8 % of the genome (Fig. 1). The most represented are three superfamilies: TC1/mariner (including mariner, MER2-Tigger, and Tc2), hAT (including MER1-Charlie and Zaphod), and PiggyBac (Lander et al., 2001; Brouha et al., 2003).

In recent years, the implementation of whole-genome and transcriptome analysis methods, as well as single-cell sequencing technologies, has substantially expanded our understanding of the role of MGEs in the regulation of gene expression. These elements have been reported to participate in a wide range of biological processes, both under normal physiological conditions and in pathological states. The investigation of the molecular mechanisms through which MGEs modulate gene activity and contribute to the pathogenesis of various diseases has therefore emerged as an important new direction in biomedical research. In particular, the role of MGEs has been examined in the development of oncological diseases (Chénais et al., 2015; Kong et al., 2019; McKerrow et al., 2022), atherosclerosis (Hueso et al., 2018), infectious diseases (Chen et al., 2023), and neurodegenerative disorders (Ravel-Godreuil et al., 2021; Roy et al., 2024; Zhang W. et al., 2025). Retrotransposons can influence gene expression both directly and indirectly. Direct effects occur when these elements integrate into genes and disrupt the reading frame, leading to premature termination of protein synthesis. Indirect effects include the provision of novel promoters, splice sites, and transcription factor binding sites. In addition, MGEs may serve as sources of enhancer and promoter sequences, influence the three-dimensional architecture of chromatin, and contribute to the emergence of new regulatory genes, including non-coding RNAs and transcription factors (Bourque et al., 2018).

Accumulating evidence suggests that MGEs have acted as key drivers of evolutionary changes underlying the emergence of placental pregnancy in mammals (Lynch et al., 2015). In this context, it is of particular interest to extend the analysis of the literature beyond somatic structures of the reproductive system to include the placenta – a unique transient organ that forms during pregnancy. Studies of the placental methylome have shown that certain repetitive elements, such as LINE-1 and human endogenous retroviruses (HERVs), function as key regulators of gene expression in placental tissue. Their methylation in the placenta is believed to regulate placentaspecific functions. For example, some retrotransposons act as alternative promoters for placenta-specific transcripts, including KCNH5 and IL2RB. The expression of an alternative form of KCNH5 may contribute to the process of trophoblast invasion.

Several studies have also investigated the activity of the LINE-1 retrotransposon during the first trimester of pregnancy (Vasilyev et al., 2021; Demeneva et al., 2023). However, despite significant advances in understanding the role of MGEs in various pathological conditions, their contribution to the development of diseases of the reproductive system remains insufficiently explored to date.

## Overview of methods for studying the impact of mobile genetic elements on the reproductive system

The reproductive system represents a unique target for the activity of mobile genetic elements due to the intensive processes of cell division, recombination, and epigenetic reprogramming that occur within it. Investigating their influence requires an integrated methodological approach combining classical genetic techniques with modern high-throughput sequencing technologies and bioinformatic analysis. Contemporary studies examining the impact of mobile genetic elements on reproductive function can be broadly divided into three key methodological approaches, each characterized by specific advantages and limitations.

Genomic and transcriptomic approaches enable the precise identification of MGE insertions and the assessment of their activity. For example, whole-genome analysis has revealed 88 tumor-specific LINE-1 insertions in high-grade serous ovarian carcinomas, which were associated with poorer patient survival (Nguyen et al., 2018).

Transcriptomic analysis (RNA-Seq), combined with specialized bioinformatic algorithms such as TEtranscripts, REdiscoverTE, SalmonTE, ExplorATE, and SQuIRE, makes it possible to quantitatively assess MGE expression and identify its association with the development of pathological conditions (Grow et al., 2015; Bourque et al., 2018).

Epigenetic approaches focus on the mechanisms controlling MGE activity, primarily DNA methylation and histone modifications. Using bisulfite sequencing, it has been demonstrated that during spermatogenesis in mice, extensive demethylation followed by remethylation of LINE-1 promoters occurs, creating a window of vulnerability for retrotransposition (Molaro et al., 2011). In humans, age-related decreases in LINE-1 methylation in sperm have been shown to correlate with reduced sperm quality (Jenkins et al., 2014). ChIP-seq studies in mouse models have confirmed the critical role of the PIWI pathway, demonstrating that piRNA-associated proteins (e. g., MIWI2) direct the establishment of the repressive H3K9me3 mark at MGE loci in germ cells. Knockout of the corresponding genes leads to widespread reactivation of transposons, disruption of meiosis, and sterility (Di Giacomo et al., 2013)

Functional studies in vitro and in vivo aim to establish causal relationships between genetic alterations resulting from the insertion of mobile genetic elements and the subsequent development of phenotypic traits. Cellular models, such as human embryonic stem cells (ESCs) equipped with reporter systems, allow real-time monitoring of LINE-1 retrotransposition and the assessment of the effects of various factors on this process (Macia et al., 2017). An example of functional in vivo validation is provided by mouse models with knockout of genes involved in the PIWI pathway (e. g., Mov10LINE-1), in which derepression of MGEs, arrest of spermatogenesis, and complete sterility have been observed (Zheng, Wang, 2012).

However, despite significant progress, the study of MGEs remains associated with several methodological challenges. In particular, the accurate analysis of repetitive sequences using short-read sequencing technologies continues to present difficulties, although improved algorithms are being developed to address this issue and reduce the occurrence of false-positive results (Gardner et al., 2017).

## Molecular mechanisms of the pathogenic impact of mobile genetic elements on the human genome

Mobile genetic elements exert multifaceted effects on the structure and function of the genome. The pathogenic potential of MGEs is realized through a variety of molecular mechanisms that disrupt genomic stability, normal gene expression, and epigenetic regulation. These mechanisms can be broadly categorized into three main groups: insertional mutagenesis, induction of genomic instability, and disruption of the epigenetic landscape (Chénais et al., 2015; Goodier et al., 2016).

Insertional mutagenesis. The most common mechanism of pathogenesis is insertional mutagenesis – the integration of a new MGE copy into a functionally significant region of the genome. More than 120 cases of hereditary diseases caused by de novo or inherited pathogenic MGE insertions have been described (Callinan, Batzer, 2006; Hancks, Kazazian, 2016). Inactivation of the target gene generally occurs via two principal mechanisms: direct disruption of the coding sequence or interference with RNA processing. In the first case, insertion of an MGE into an exon disrupts the open reading frame, often resulting in the formation of a premature termination codon and subsequent degradation of the transcript through nonsense-mediated mRNA decay (NMD) (Miki et al., 1992). In the second case, insertion into intronic or exonic regions may create cryptic splice sites, leading to aberrant pre-mRNA splicing, inclusion of pseudoexons, or exon skipping, and consequently to the synthesis of defective protein isoforms. A striking example of this mechanism is the insertion of an SVA element into the 3ʹ untranslated region (3ʹ-UTR) of the FKTN gene, which is associated with Fukuyama muscular dystrophy. This insertion does not cause complete knockout inactivation of the gene but instead induces alternative splicing, resulting in the synthesis of a C-terminally truncated protein. This truncated protein accumulates in the Golgi apparatus and the endoplasmic reticulum, disrupting post-translational modification processes and thereby contributing to disease development (Taniguchi-Ikeda et al., 2011). In addition, the process of retrotransposition may be accompanied by deletion at the integration site. The insertion of retrotransposon cDNA (LINE-1, Alu, SVA) is associated with the formation of doublestrand DNA breaks, which can lead to the loss of sequences ranging from several base pairs to several megabases. Such de novo deletions associated with MGE insertions have been identified in human genomes and may directly eliminate functionally important genetic elements (Gilbert et al., 2002; van den Hurk et al., 2007).

The induction of genomic instability and chromosomal rearrangements. The second mechanism underlying the pathogenicity of MGEs is the induction of genomic instability and chromosomal rearrangements. The high degree of sequence homology among numerous copies of MGEs (e. g., Alu repeats) creates a substrate for non-allelic homologous recombination. Incorrect pairing of homologous sequences located at different genomic loci during meiosis can lead to deletions, duplications, inversions, or translocations (Sen et al., 2006). This mechanism underlies several microdeletion syndromes, such as hereditary neuropathy with liability to pressure palsies (HNPP), which is caused by reciprocal recombination between repeated sequences in the 17p11.2–12 region of chromosome 17, encompassing the gene encoding peripheral myelin protein 22 (PMP22) (Polynnikova et al., 2021).

Disruption of epigenetic regulation represents the third mechanism of the pathogenic effects of MGEs. This mechanism is associated with the ability of MGEs to alter the normal DNA methylation status of genomic regions. Since the transcriptional activity of MGEs poses a potential threat to cellular integrity, eukaryotic cells have evolved mechanisms to repress them, primarily through methylation of CpG dinucleotides within their promoter regions (Figueiredo et al., 2009). It has been reported that the insertion of MGEs may also influence adjacent DNA regions. Highly methylated promoters of integrated MGEs (such as LINE-1 or SVA) can serve as targets for the recruitment of DNA methyltransferases and histonemodifying complexes, leading to the spread of heterochromatin into nearby regulatory regions of genes and resulting in their transcriptional silencing (Slotkin, Martienssen, 2007).

## Why is the reproductive system particularly vulnerable?

The human reproductive system exhibits increased susceptibility to dysregulation of mobile genetic elements due to its unique biological features, including extensive epigenetic reprogramming, evolutionary co-option of MGEs, and stringent requirements for genomic stability (Smith et al., 2012).

A key factor underlying this vulnerability is the large-scale epigenetic reprogramming that occurs during gametogenesis and early embryogenesis. This process involves global DNA demethylation, which is necessary to erase parental epigenetic marks and establish totipotency (Smith et al., 2012). However, the transient removal of repressive methylation marks, particularly at MGE promoter regions such as LINE-1 and Alu, creates a temporary window of vulnerability. During this period, the transcriptional potential of MGEs is reactivated, substantially increasing the risk of de novo insertional mutagenesis in genes critical for gamete and embryonic development (van den Hurk et al., 2007; Grow et al., 2015). In this way, the very biological program that ensures totipotency and developmental progression also elevates genomic instability.

It should be noted that several studies indicate that a certain level of MGE activity is physiologically necessary for normal reproductive function. Throughout evolution, MGE sequences have been co-opted into regulatory networks controlling embryogenesis and placentation (Lynch et al., 2015). For instance, the expression of specific retrotransposon families – MERVL in mice and HERVH in humans – is required for activation of the totipotency genetic program at the 2-cell embryonic stage, functioning as alternative enhancers and promoters (Macfarlan et al., 2012; Grow et al., 2015). Furthermore, evidence suggests that some proteins mediating cytotrophoblast fusion and the formation of the syncytiotrophoblast in the placenta are of retroviral origin. For example, syncytin genes, originally derived from the envelope (env) genes of endogenous retroviruses (HERV-W, HERV-FRD), are essential for proper placental development (Mi et al., 2000; Blaise et al., 2003).

At the same time, disruption of the epigenetic control mechanisms regulating MGEs can lead to uncontrolled transcription and retrotransposition, resulting in damage to genes essential for reproduction (Vasilyev et al., 2021). Such dysregulation is thought to be associated with a broad spectrum of reproductive pathologies, including idiopathic infertility, implantation failure, pregnancy loss, and complications related to placental dysfunction, such as preeclampsia and fetal growth restriction. The placenta is particularly vulnerable because, unlike somatic tissues, it maintains global hypomethylation of the genome, including LINE-1 promoters (He, Ecker, 2014). Under normal conditions, this hypomethylated state supports the unique functions of the trophoblast; however, aberrant hyperactivation of retrotransposons can impair trophoblast invasiveness and spiral artery remodeling, directly contributing to the pathogenesis of preeclampsia and fetal growth restriction (Vasiliev et al., 2015).

## MGEs in the pathogenesis of gametogenesis disorders and infertility

Dysregulation of mobile genetic elements, particularly LINE-1 retrotransposons, represents a key molecular mechanism associated with impaired fertility and gametogenic pathology. The pathogenic effects are mediated through disruption of epigenetic control, induction of genomic instability, and aberrant regulation of genes critical for reproduction.

Epigenetic dysregulation of MGEs in germ cells. Unlike somatic cells, where LINE-1 promoters are constitutively hypermethylated, the epigenetic control of these elements in germ cells is highly dynamic and tissue-specific. One major risk factor for impaired gamete maturation is age-dependent alteration of LINE-1 methylation status. In spermatozoa, LINE-1 promoter methylation increases with paternal age (Jenkins et al., 2014), whereas in human oocytes at the diplotene stage and in ovulated secondary oocytes, LINE-1 elements exhibit relative hypomethylation (Smith et al., 2012). Such epigenetic imbalance creates conditions for reactivation of MGE transpositional activity, potentially leading to insertional mutagenesis in key genes that regulate meiosis, gamete differentiation, and early embryonic development. Studies in mice have shown that spermatozoa from individuals with idiopathic infertility exhibit significant hypomethylation of LINE-1 promoters compared to fertile controls (Jachowicz et al., 2017). This epigenetic disruption is accompanied by elevated transcriptional and retrotranspositional activity of LINE-1. Notably, excessive LINE-1 expression correlates with increased sperm DNA fragmentation, indicating a direct link between retrotransposon derepression and genomic damage in male gametes. LINE-1 hypomethylation thus serves as an independent prognostic marker of impaired spermatogenesis and reduced ejaculate quality (Jenkins et al., 2014).

Insertional mutagenesis in genes critical for spermatogenesis. The pathogenic impact of mobile genetic elements on male reproductive function is primarily mediated through insertional mutagenesis. It has been demonstrated that de novo LINE-1 retrotransposon insertions can disrupt the integrity of genes essential for normal spermatogenesis. Of particular significance are lesions within the AZF (Azoospermia Factor) locus of the Y chromosome, which contains a cluster of genes directly responsible for sperm production. LINE-1 insertions in DAZ (Deleted in Azoospermia) gene family members located within the AZF region have been identified as a direct cause of gene inactivation, leading to impaired spermatogenesis and the development of azoospermia (Hancks, Kazazian, 2016).

Beyond effects on Y-chromosome genes, retrotranspositional activity can also impact critical autosomal loci. For instance, LINE-1 insertions in the FKBP6 gene, which encodes a protein involved in meiotic division, have been reported. Mutations in FKBP6 are associated with defects in chromosome pairing and synaptonemal complex formation during prophase I of meiosis, ultimately resulting in male infertility (Wyrwoll et al., 2022).

Disruption of early embryogenesis and implantation. The second wave of extensive epigenetic reprogramming, which begins after fertilization and continues until the blastocyst stage, is accompanied by passive genome-wide demethylation and a marked increase in the transcriptional potential of LINE- 1 elements. Under normal conditions, this process is tightly regulated by RNA interference mechanisms and is essential for chromatin remodeling and activation of the embryonic genome (Jachowicz et al., 2017). However, disruption of the delicate balance between MGE activation and repression can lead to pathological outcomes. It has been reported that artificial suppression of LINE-1 transcription in mouse zygotes impairs cleavage and arrests development at early stages (Percharde et al., 2018). This is because LINE-1 sequences act as alternative promoters for genes critical for cell division (e. g., TP53) and participate in the regulation of higher-order chromatin organization (Chow et al., 2010).

## Pregnancy loss associated with chromosomal abnormalities

An important aspect of reproductive pathology is the interplay between MGE activity and chromosomal instability. Aneuploidy, in turn, can induce global changes in the methylome It has been shown that in placental tissues with mosaic forms of aneuploidy, LINE-1 methylation levels are significantly increased (Vasiliev et al., 2015). This may reflect a compensatory mechanism, whereby promoter hypermethylation reduces transcriptional load on the genome under conditions of disrupted gene dosage balance.

Moreover, MGE activation in cases of pregnancy loss may also be secondary, serving as a marker of general cellular stress. Oxidative stress, which is characteristic of placentas with chromosomal abnormalities, induces global DNA demethylation, relieving repression of LINE-1 and Alu promoters (Lou et al., 2020). Subsequent retrotransposition and accumulation of double-strand DNA breaks further destabilize the trophoblast genome, reinforcing the pathological state and rendering continued pregnancy development unviable (Garcia-Perez et al., 2016). Thus, MGE dysregulation acts not only as a trigger but also as a universal amplifier of pathological processes, contributing to reproductive failure at the earliest stages of gestation

## The contribution of MGEs to the pathogenesis of preeclampsia

Previous studies have shown that hundreds of genes in the endometrium gain or lose expression coinciding with the onset of pregnancy and decidualization in mammals (Lynch et al., 2015; Mika et al., 2021). To prepare the uterus for embryo implantation, the endometrium undergoes decidualization, during which stromal cells transform into decidual stromal cells (DSCs). The decidual layer formed as a result of this process supports embryo implantation and protects the fetus from maternal immune rejection. Decidualization is regulated by progesterone through its receptor (PGR), the secondary messenger cAMP, protein kinase A (PKA), and the transcription factor FOXO1 (Kajihara et al., 2013). This evolutionarily acquired process in eutherian mammals is accompanied by extensive changes in gene regulatory activity, cellular organization, and endometrial physiology, all of which are essential for successful implantation and pregnancy (Mess, Carter, 2006; Kin et al., 2014).

MGEs are believed to have contributed substantially to the development and function of DSCs during this adaptive process. Two major waves of transposon activity and genomic integration have remodeled the transcriptome and regulatory landscape of DSCs. Current evidence indicates that genes located near regulatory elements derived from transposons exhibit high progesterone sensitivity and appear to play key roles in mediating the hormone’s signaling response, providing binding sites for progesterone receptors (Mika, Lynch, 2022). Defective decidualization during early pregnancy can lead to fetal growth restriction, pregnancy loss, or obstetric complications such as preeclampsia (PE).

Preeclampsia is one of the most severe pregnancy complications, characterized by defective placentation, endothelial dysfunction, and a systemic inflammatory response. The central pathogenic hypothesis of PE is incomplete trophoblast invasion of the uterine spiral arteries, resulting in placental hypoxia. The role of MGEs – such as LINE-1, Alu, and SVA elements – in regulating gene expression and epigenetic mechanisms in decidual cells remains insufficiently explored.

Preeclampsia is accompanied by systemic inflammation, in which decidual cells play a pivotal role through the secretion of pro-inflammatory cytokines. Studies have shown that PSG1, PSG6, and PSG11 increase secretion of anti-inflammatory cytokines IL-10 and IL-6, while all PSG genes activate the TGFB receptor in immune cells (Warren et al., 2018). MGE activation, particularly LINE-1, may induce inflammatory pathways via the production of double-stranded RNA (dsRNA), which is recognized by innate immune receptors such as TLR3. Similar mechanisms are observed in autoimmune diseases, where transposons trigger an interferon response (Crow, 2014). However, direct evidence supporting this mechanism in PE pathogenesis within decidual cells remains limited and requires further investigation. Integration of transposons into the human genome has also influenced the regulation of key placental hormones, including leptin (LEP), insulin-like protein 4 (INSL4), and corticotropin-releasing hormone (CRH), highlighting the significant role of MGEs in modulating processes critical for pregnancy development.

Furthermore, some studies suggest that hyperactivation of MGEs can directly impair trophoblast differentiation and function. Successful placentation requires cytotrophoblast differentiation along two main pathways: into syncytiotrophoblast and invasive extravillous trophoblast. Disruption of this finely tuned differentiation process, resulting in incomplete extravillous trophoblast invasion and failure to remodel spiral arteries, is directly associated with PE development. LINE-1 and other retrotransposons have been shown to interfere with key signaling pathways controlling trophoblast differentiation, which may underlie the placentation defects central to the pathogenesis of preeclampsia (Demeneva et al., 2024).

## Mobile genetic elements and their role in the pathogenesis of fetal growth restriction

Fetal growth restriction (FGR), also referred to as intrauterine growth restriction, is defined as the birth of a fetus with a body weight and/or length at or below the 10th percentile, or ≥2 standard deviations below the mean for a given gestational age (Li et al., 2019). FGR is associated with an increased risk of perinatal mortality, neurological deficits, delayed psychomotor development, metabolic syndrome, and cardiovascular diseases in adulthood (Entringer et al., 2012). The pathogenesis of FGR is multifactorial, involving maternal, placental, and fetal determinants, among which dysregulation of mobile genetic elements plays a significant role.

LINE-1 elements are the most abundant autonomous retrotransposons in the human genome. Under normal conditions, LINE-1 sequences are deeply methylated and transcriptionally silenced in somatic tissues of adults (Figueiredo et al., 2009). However, under conditions of epigenetic instability – such as methyl donor deficiency – promoter hypomethylation can occur, leading to LINE-1 activation, transcription, and retrotransposition (Howard et al., 2008). Folic acid, as a key methyl group donor, plays a central role in maintaining global DNA methylation. In vitro studies have shown that culturing mouse embryonic stem cells (mESCs) under folate-deficient conditions induces intracellular depletion of 5-methyltetrahydrofolate, reduces levels of S-adenosylmethionine (SAM), and subsequently leads to LINE-1 hypomethylation (Chang et al., 2013). Similar observations have been made in vivo: pregnant women carrying fetuses with neural tube defects (NTDs) exhibited significantly reduced LINE-1 methylation, which correlated with low maternal serum folate levels (Li et al., 2019).

Mouse model studies have demonstrated that LINE-1 hypomethylation and activation in the placenta correlate with reduced fetal mass and disrupted placental architecture, including thinning of the syncytiotrophoblast layer and decreased vascularization. In human cohort studies, LINE-1 methylation levels in cord blood and placental tissue of infants with FGR were significantly lower than in infants with normal birth weight. Moreover, mothers with low folate levels during the first trimester exhibited an increased risk of giving birth to infants with FGR, accompanied by reduced LINE-1 methylation in the placenta (Joubert et al., 2016; Li et al., 2019).

## Oncogenic role of mobile genetic elements

A review of experimental studies highlights the significant role of mobile genetic elements, particularly LINE-1 retrotransposons, in the pathogenesis of malignancies of the reproductive system. In epithelial ovarian cancer, team of S. Sato (Sato et al., 2023) demonstrated that the LINE-1 ORF1p protein is not only expressed but also secreted by tumor cells, detectable in patient plasma and ascitic fluid, suggesting its potential as a biomarker. Earlier, a whole-genome analysis by the group of T.H.M. Nguyen (Nguyen et al., 2018) identified 88 tumorspecific LINE-1 retrotransposition events in high-grade serous ovarian carcinomas, which correlated with poorer patient survival. In vitro experiments showed that inhibiting LINE-1 expression reduced cell invasion and migration while increasing apoptosis (Fu et al., 2023). In endometrial cancer, significant hypomethylation of LINE-1 promoters was observed in tumor tissues compared to normal endometrium, with the most pronounced hypomethylation in papillary serous carcinomas. This epigenetic derepression led to increased LINE-1 transcription, particularly in p53-mutated tumors with high levels of copy number variation (CNV), suggesting a synergistic effect between p53 mutation and MGE activation (Zhang B. et al., 2014). The research group of W. McKerrow further confirmed that LINE-1 overexpression can induce phosphorylation of RAD50, a key step in the DNA double-strand break response. Statistical analysis revealed a strong correlation between LINE-1 activity, chromosomal aberrations, and replication stress pathway activation (McKerrow et al, 2022). In cervical cancer, RNA-seq analyses of invasive carcinoma samples showed that the highest number of differentially expressed retroelements was associated with co-infection by multiple HPV types, indicating a potential synergistic effect in disease progression (Curty et al., 2021). In prostate cancer, systemic analysis of whole-genome sequencing data revealed significantly higher somatic retrotransposition in metastases compared to primary tumors. Notably, 68 % of these insertions localized to exons, introns, or regulatory regions of genes, including tumor suppressors, directly implicating MGEmediated inactivation of protective pathways and enhanced genomic instability (Tubio et al., 2014).

These studies, although compelling, represent only a fraction of an actively expanding research field. Further investigations are required to fully elucidate the regulatory networks controlling MGE activity in different tissues and cell types, as well as to evaluate the contribution of other retrotransposon families to oncogenesis.

## Conclusion

**Fig. 2.**
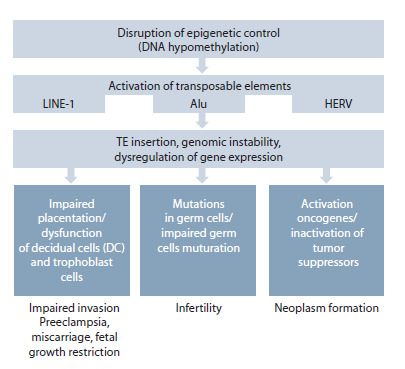
Key pathways of MGE dysregulation and their contribution to the pathophysiology of reproductive disorders.

This review underscores the fundamental role of mobile genetic elements in regulating physiological processes of the reproductive system and their involvement in a broad spectrum of pathological conditions. Among various MGE families, LINE-1 retrotransposons have been studied most extensively. Epigenetic dysregulation, particularly promoter hypomethylation during physiological reprogramming or in response to cellular stress, is a primary trigger for MGE-mediated pathogenic effects. This dysregulation activates LINE-1 transcription and retrotransposition, initiating a cascade of events that includes insertional mutagenesis, induction of genomic instability, and disruption of normal gene regulation through the emergence of alternative promoters (Fig. 2).

Collectively, these molecular disturbances contribute to severe obstetric complications. Preeclampsia and fetal growth restriction arise from impaired trophoblast differentiation and invasion of spiral arteries, processes closely associated with LINE-1 hyperactivation. Pregnancy loss occurs due to the combined effects of insertional mutagenesis, chromosomal instability, and defects in placental development. Future progress in this rapidly evolving field will benefit from advances in methodology. Long-read sequencing technologies enable direct detection of full-length MGE insertions in complex genomic regions (de la Morena-Barrio et al., 2022; Yano et al., 2024). Integrative multi-omics approaches combining ATACseq and RNA-seq will allow analysis of MGE influence on three-dimensional chromatin architecture, while CRISPR-Cas genome editing provides unique opportunities to model the effects of specific MGE insertions or deletions in functional studies. Thus, investigating MGEs offers a promising avenue in molecular biology and genetics, enhancing our understanding of mechanisms underlying reproductive system homeostasis and laying the foundation for the development of diagnostic and therapeutic strategies for reproductive disorders

## Conflict of interest

The authors declare no conflict of interest.
